# Masquelet’s induced membrane technique in the upper limb: a systematic review of the current outcomes

**DOI:** 10.1186/s10195-024-00815-w

**Published:** 2025-01-27

**Authors:** Davide Pederiva, Lapo De Luca, Cesare Faldini, Luigi Branca Vergano

**Affiliations:** 1 Unità Operativa di Ortopedia e Traumatologia, APSS Trento, Largo Medaglie d’oro, 9, 38121 Trento, Italy; 2 IRCCS Rizzoli Orthopedic Institute, Bologna, Italy

**Keywords:** Masquelet, Induced membrane, Upper limb, Bone defect

## Abstract

**Background:**

The Masquelet induced membrane technique is a surgical procedure that allows the reconstruction of segmental bone defects using a relatively simple approach that requires minimal resources from both the healthcare facility and the patient. Historically applied to the lower limb, this technique is gaining increasing attention in the literature for its use in the upper limb.

**Methods:**

A systematic review of the literature was conducted using the PubMed and Google Scholar databases to identify all studies reporting the outcomes of the Masquelet induced membrane technique in the long bones of the upper limb (humerus, radius, and ulna) with a sample size of at least 3 patients. The papers had to include the length of the bone defect, a description of the protocol used for treatment, the complications of each case, and the anatomical location of the defect. The studies that did not meet the above inclusion criteria were excluded.

**Results:**

The search identified 1044 studies, of which 15 met the inclusion criteria. These studies described a total of 156 patients with a mean age of 42 years. The affected bone segments included the humerus in 22 cases and the forearm in 134 cases. In 108 cases, the bone defect was septic. The average defect length was 4.5 cm. PMMA was used as a spacer in all cases, with antibiotics added in 77% of them. The average time interval between the first and second phases of the procedure was 9.5 weeks, and bone union took an average of 5.5 months. The mean follow-up duration was 48 months, and the complication rate was 21%, ranging from 0% to 75%.

**Conclusions:**

The Masquelet induced membrane technique is a viable surgical option for managing segmental bone defects of the upper limb. However, the complication rate remains significant. Further research is needed to identify strategies to improve the outcomes of this technique.

*Level of Evidence*: Level 2.

## Introduction

Managing segmental bone defects represents one of the most complex challenges in orthopedics and traumatology, with significant implications for patients’ quality of life [1]. These defects can result from trauma, infections, tumor resections, or other pathologies, and their treatment requires a multidisciplinary and personalized approach [2]. Over the years, various therapeutic strategies have been developed to address this issue, each with specific advantages and limitations.

Traditionally, bone defects were filled with autologous bone grafts, which still represent the gold standard for bone regeneration. Autologous cancellous bone grafts, whether harvested from the iliac crest or via reamer–irrigator–aspirator (RIA), primarily serve as an osteoconductive substrate with osteogenetic and osteoinductive properties [3]. However, this strategy has three significant limitations. The amount of bone that can be harvested is limited [4], associated complications are non-negligible [5]; and the bone defect that needs to be filled must be less than 5 cm in length, as larger defects would not heal due to physiological graft resorption [6, 7].

When primary grafting of the bone defect is likely to fail, alternative therapeutic strategies are available. Acute shortening of up to 5 cm is an option for the upper limb but is seldom accepted by the patient [1]. Distraction osteogenesis has shown a high success rate in managing bone defects [8], but it comes with a prolonged reconstruction time, which is why its indication in the upper extremity is very limited [9]. Vascularized fibula grafting is another therapeutic solution [10], but it requires high microsurgical expertise and is associated with considerable donor-site morbidity [11].

In this context, the induced membrane technique (MIMT) described by Masquelet and Begue [6] is particularly relevant. This technique involves reconstructing the bone defect through two well-defined phases [6]: an initial phase of thorough debridement of non-viable tissues and of filling the defect with a spacer, followed by a second phase where the spacer is removed and the defect is filled with bone graft while preserving the induced membrane. The potential advantages of MIMT compared to the aforementioned techniques are numerous [12, 13]: it does not require specialized tools or high costs, it involves less complex surgery, the interval between the two phases allows optimal management of soft tissues, it does not demand significant patient compliance, and the time required for bone consolidation is independent of the defect size.

Despite the documented successes of the Masquelet technique in the lower limbs, its adoption in the upper limb is less common and less studied. Anatomical differences, the functional complexity of the upper limb, and the need to preserve joint mobility present challenges that make the application of the technique particularly complex. However, the potential of this approach is promising, and there is a growing interest in extending its use to this anatomical region, as evidenced by the significant increase in publications on the topic over the past 5 years.

The most recent systematic review [2] of the literature on the use of the Masquelet technique in the upper limb dates back to studies published before 2019 and indiscriminately considered all upper-limb bones without differentiating between the humerus and forearm versus the clavicle and metacarpals. Since we believe that therapeutic choices for these regions differ, even just due to size factors, and given that the few published studies on the topic are case reports and case series, this review was designed to update the literature regarding the outcomes and complications associated with the use of the Masquelet technique in managing segmental defects of the long bones in the upper limb.

## Methods

### Literature search

The medical databases PubMed and Google Scholar were searched on 22 May 2024 for relevant publications on the use of the Masquelet induced membrane technique in the management of bone defects in the upper limb. The databases were filtered for studies published in English between 1 January 1990 and 21 May 2024. The following MeSH (Medical Subject Headings) terms were used: “induced membrane technique” OR “Masquelet technique” AND “upper limb” OR “upper extremity.”

### Inclusion and exclusion criteria

Full-text, peer-reviewed articles describing the use of the induced membrane technique in the long bones of the upper limb, specifically the humerus and forearm, with a sample size of at least 3 patients were included. Publications needed to include the length of the bone defect, the description of the treatment protocol used, the outcomes and complications of each case, and the anatomical location of the defect. Studies not meeting these inclusion criteria were excluded.

The articles were initially screened using titles and abstracts to identify those meeting the inclusion criteria. Relevant articles were then selected for full-text analysis according to our inclusion and exclusion criteria. The PRISMA (Preferred Reporting Items for Systematic Reviews and Meta-Analyses) [14] guidelines were followed, and a flow diagram was created to summarize the process of including the analyzed studies.

### Data extraction

The selected studies were analyzed, and relevant information was extracted into a database created using Microsoft Excel for Mac (Microsoft Corporation, Redmond, WA, USA).

The level of evidence and quality of each study were assessed using the methodological index for non-randomized studies (MINORS) [15].

The following data were recorded, if available: number of patients treated, age, mechanism of segmental bone loss, anatomical site, defect size, time between spacer placement and grafting, type of spacer, time to bone union, follow-up duration, and complications.

### Outcome measures

The primary outcome was the bone union time using the induced membrane technique in the long bones of the upper limb. The secondary outcome was the complication rate.

### Statistical analysis

The collected data were analyzed using descriptive statistics. Continuous variables were reported as means with ranges (minimum–maximum), while categorical variables were expressed in terms of frequencies and percentages.

The time required for bone union was categorized based on defect length: ≤ 2 cm, between 2 and 5 cm, and > 5 cm. A Kruskal–Wallis test was then performed to determine if increasing defect size was associated with prolonged bone-union time.

For the secondary outcome, the presence of complications was analyzed using the chi-square test with Yates’ correction to check whether the complication rate differed between the humerus and forearm.

The level of statistical significance was set at* p* < 0.05.

## Results

### Search results

**Fig. 1:**
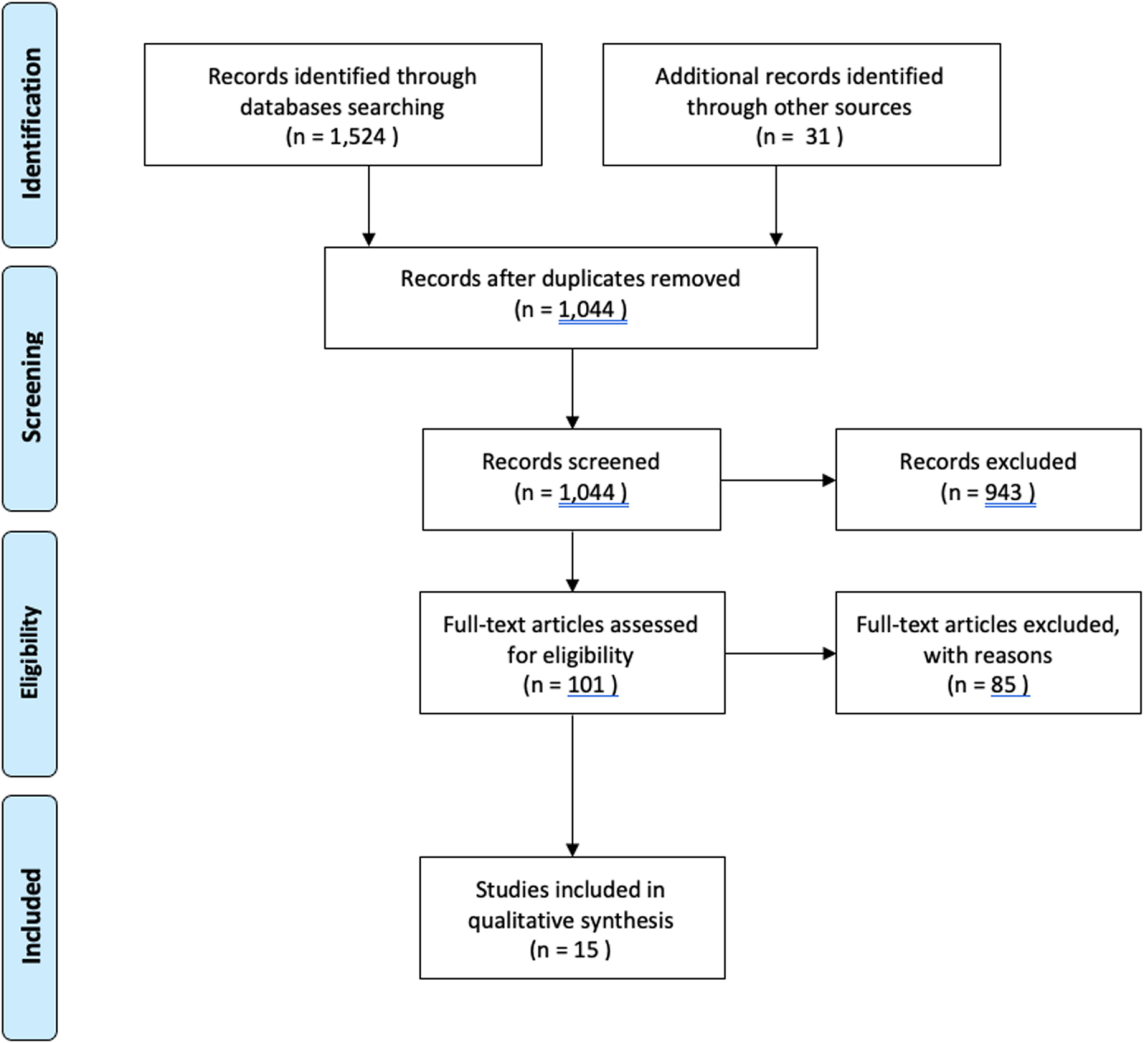
PRISMA flowchart showing the search for and selection of articles

The study selection process is summarized in Figure 1 using a PRISMA flow diagram. The initial search yielded 1524 articles. After removing duplicates, a total of 1044 records remained. Based on the title and abstract, 943 studies were excluded as they were deemed irrelevant to this study. The remaining 101 papers were assessed in detail to verify their alignment with the inclusion criteria. After full-text screening, a total of 15 studies met the inclusion criteria.

### Quality assessment of studies

All selected studies were retrospective case series of a medium-to-small size (sample size = 3–32) that reported the outcomes of using the Masquelet technique in the long bones of the upper limb.

The manuscripts were published between 2014 and 2023. According to the MINORS scoring system, one study scored 6/16 points, two studies scored 7/16 points, two studies scored 8/16 points, four studies scored 9/16 points, two studies scored 10/16 points, three studies scored 11/16 points, and one study scored 12/16 points (Table 1).

**Table 1:** List of included studies

Authors	Year	Country	Methodological score
Wong et al. [16]	2014	China	9
Kawakami et al. [17]	2015	Japan	9
Luo et al. [18]	2016	USA	8
Giannoudis et al. [19]	2016	UK	12
Anoumou et al. [20]	2017	Ivory Coast	9
Walker et al. [21]	2019	USA	10
Dhar et al. [22]	2019	India	7
Gaillard et al. [23]	2020	France	10
Bourgeois et al. [24]	2020	France	11
Commeil et al. [25]	2021	France	8
Lauthe et al. [26]	2021	France	11
Rougereau et al. [27]	2021	France	6
El Farhaoui et al. [28]	2022	Morocco	7
Ma et al. [29]	2022	China	9
Braswell et al. [30]	2023	USA	11

### Cohort characteristics

The studies included 156 patients (Table 2) with a mean age of 42 years (ranging from 1 year to 79 years); the mean follow-up was 48 months (5–144 months).

**Table 2:** Summary of results

Author	Numerosity	Age (years)	Location	Cause	Type	Defect size (cm)	Spacer	Antibiotic	Second phase (weeks)	Graft	Union time (months)	Follow-up (months)	Complications %
Wong et al. [16]	3	66	1H, 2U	1O, 2C	2INF, 1BL	3	PMMA	Gen, Van	7	ICG	–	–	0
	2M, 1F	(60–79)				(2–4)			(7–8)				
Kawakami et al. [17]	4	43	1H, 3RU	4O	4INF	3.5	PMMA	nr	–	ICG	4	49	75
	4M	(29–59)				(2.5–5)					(3–6)	(36–72)	
Luo et al. [18]	7	47	3RU, 1R, 3U	–	7INF	5.5	PMMA	nr	–	ICG	–	87	43
	3M, 4F	(32–74)				(4.5–7)						(41–150)	
Giannoudis et al. [19]	15	50	1H, 4RU, 7R, 3U	–	6INF, 9BL	3	PMMA	Van	7	6 ICG, 9 RIA	3.5	–	7
	12M, 3F	(18–77)				(2–6.5)			(6–8)		(2–6)		
Anoumou et al. [20]	6	35	2H, 2RU, 1R, 1U	–	6INF	4	PMMA	–	13	ICG	4.5	30	50
	3M, 3F	(18–62)				(2–6)			(8–16)		(4–6)	(5–44)	
Walker et al. [21]	9	43	7R, 2U	8O, 1C	8INF, 1BL	4	PMMA	Van+Tor	6	1ICG, 8RIA	4.5	–	56
	7M, 2F	(22–62)				(2–5)			(4–8)		(3–12)		
Dhar et al. [22]	12	38	5R, 7U	–	12INF	5	PMMA	5Van, 7 Tor	6	ICG	8	–	0
	11M, 1F	(19-63)				(3.5-7)					(6–12)		
Gaillard et al. [23]	15	47	15H	3O, 12C	4INF, 8BL	2	PMMA	NO	7	13ICG/RIA, 2 NVF	4.5	24	13
	7M, 8F	(21–62)				(0–12.5)					(4–9)	(6–30)	
Bourgeois et al. [24]	6	40	3R, 3U	4O, 1C, 1T	2INF, 4BL	6	PMMA	Gen	30	3ICG, 3RIA	4	103	33
	5M, 1F	(17–78)				(5–11)			(20–55)		(2–6)	(67–144)	
Commeil et al. [25]	10	44	2RU, 1R, 7U	1O, 9C	6INF, 4BL	4	PMMA	Gen	13	ICG	9	50	10
	8M, 2F	(24–65)				(2–8)			(8–20)		(4–13)	(14–74)	
Lauthe et al. [26]	13	39	5RU, 2R, 6U	–	6INF, 7BL	4	PMMA	NO	6	12ICG, 1ICG+NVF	5	30	23
	13M	(18–67)				(0–12)					(3–8)		
Rougereau et al. [27]	3	9	1R, 2U	3T	3BL	9	PMMA	NO	29	ICG	–	102	33
	1M, 2F	(1–17)				(6–11)			(12–40)				
El Farhaoui et al. [28]	7	34	2H, 5R	3O, 4C	5INF, 2BL	3.5	PMMA	Gen	18	6ICG, 1NVF	4	38	29
	6M, 1F	(18–57)				(1.5–9)			(6–88)		(3–6)		
Ma et al. [29]	32	43	4R, 28U	22O, 10C	32INF	6	PMMA	16Van, 10Mer, 6Van+Mer	6	ICG	6	–	16
	20M, 12F	(19–62)				(3.5–8)					(4–9)		
Braswell et al. [30]	14	–	2RU, 12R	–	8INF, 6BL	4	PMMA	–	7	ICG	–	–	57
	–					(2–6)			(6–9)				
Total	156	42	22H, 21RU, 49R, 64U	–	108INF, 48BL	4.5	PMMA	–	9.5	136ICG, 17RIA, 3NVF	5.5	48	21

*H* humerus,* R* radius,* U* ulna,* O* open fracture,* C* closed fracture,* T* tumor,* INF* infection,* BL* aseptic bone loss,* Gen* gentamycin,* Van* vancomycin,* Tor* tobramycin,* Mer* meropenem,* ICG* iliac crest graft,* NVF* non-vascularized fibula

### Etiology and site of injury

The patients included in the studies had bone defects due to both septic and aseptic causes. Of the 156 patients, 108 (69%) had septic lesions, while 48 (31%) had aseptic lesions. The initial cause of the bone defect was not always described. In the nine studies where it was described (totaling 89 patients), most had an open fracture in their clinical history (46/89; 52%), followed by closed fractures (39/89; 44%), and finally, a small percentage had an oncological cause (4/89–4%).

The most frequently affected site was the forearm. Specifically, the radius was the most affected bone segment (64/156; 41%), followed by the ulna (49/156; 31%). The involvement of both the radius and ulna (21/156; 14%) was as common as the involvement of the humerus (22/156; 14%).

### Size of the bone defect and type of spacer

The average size of the defect was 4.5 cm, with a range from 0 to 12.5 cm. Most (81/156; 52%) had a defect between 2 and 5 cm in size. Next in frequency were defects larger than 5 cm but less than 10 cm (57/156; 37%), defects smaller than 2 cm (13/156; 8%), and finally, defects larger than 10 cm (5/156; 3%).

Considering the location of the bone defect, the average defect size was 2.5 cm at the humeral level, while at the forearm level, the average size increased to 5 cm.

The choice of spacer material was PMMA in 100% of cases. The decision to add antibiotics to the cement was reported in 13 studies. Two studies reported adding antibiotics without specifying which, three studies used gentamicin, one study used vancomycin, one study used a combination of vancomycin and gentamicin, one study used either vancomycin or tobramycin, one study used a combination of vancomycin and tobramycin, one study used vancomycin and meropenem either independently or combined, and finally, in three studies, no antibiotics were added to the cement. Overall, antibiotics were added in 77% (105/136) of cases. The most commonly used antibiotic (either alone or combined) was vancomycin (54/125; 43%), followed by gentamicin (26/125; 21%).

### Time interval and type of graft

The average time between spacer placement and its replacement with bone graft was 9.5 weeks. This parameter, reported in 13/15 studies, showed considerable variability from study to study, with average values ranging from 6 to 29 weeks.

The type of graft used was almost entirely autologous iliac crest graft (136/156; 87%), followed by RIA (17/156; 11%) and autologous non-vascularized fibula graft (3/156; 2%).

### Bone union time and follow-up

The bone union time was reported in 11 studies. The global mean value was 5.5 months, but there was considerable variability from study to study (average range from 3.5 to 9 months).

When categorized based on the size of the bone defect, the mean time to union was 4.5 months for defects equal to or less than 2 cm, 6.5 months for defects between 2 and 5 cm, and 7 months for defects larger than 5 cm. These values were not significantly different (*p* = 0.112).

The mean follow-up duration, reported in nine studies, was 48 months. The difference between studies was considerable, with average follow-up values ranging from 24 to 102 months.

### Complications

Overall, the complication rate was 21% (33/156), with a reported range from 0 to 75%. Two studies reported no complications.

The most frequent complication was infection (9/33–27%), which required one or more superficial debridement procedures in 5 cases, graft renewal in 1 case, and revision with non-vascularized fibula graft in 1 case; there was a failure to consolidate in 1 case and amputation of the limb after multiple salvage attempts in 1 case.

The need to revise the graft for non-septic causes occurred in 4 cases (12%). In 1 case, two additional iliac crest grafts with added demineralized bone matrix were necessary; in 1 case, an additional iliac crest graft was required following initial RIA; and the remaining 2 cases were managed with additional iliac crest grafts.

Aseptic failure of consolidation was described in 5 cases (15%). Of these, two were revised with a non-vascularized fibula graft after multiple attempts with additional grafts, resulting in consolidation in 1 of the 2 cases.

Overall, 6 cases (6/156–4%) did not achieve bone union.

Additional complications included adhesion formation or stiffness (7/33); the development of algodystrophy (1/33); seroma formation (1/33); the need for ulna shortening (2/33); irritation related to fixation devices, leading to their revision or removal (2/33); peripheral nerve paralysis (2/33); and peri-implant fracture (1/33).

When complications were divided based on anatomical site (4/22 (18%) at the humeral level and 29/134 (22%) at the forearm level), the difference was not statistically significant (*p* = 0.931).

## Discussion

The Masquelet induced membrane technique has steadily established its role as an effective and efficient approach to managing segmental bone defects [12, 13]. Initially limited, its use has gradually expanded to include the long bones of the upper limb [2]. This study highlighted a 96% success rate in bone union (with failure in only 6 out of 156 patients) and a 21% complication rate. The average bone union time was 5.5 months, with an average bone defect length of 4.5 cm.

This review reported an average bone union time of 5.5 months, with a range of 3.5 to 9 months. There was no significant difference between the union time and the length of the bone defect. However, a trend was observed that suggests faster healing for defects smaller than 2 cm (4.5 months) compared to those between 2 and 5 cm or larger than 5 cm (6.5 and 7 months, respectively). These findings confirm the current evidence in the literature [1, 12, 13] in two main aspects: smaller defects (≤ 2 cm in our study) heal faster than larger ones, and the healing time for defects larger than 2 cm seems independent of the defect size.

Although questions may arise regarding the role of the induced membrane technique in bone defects smaller than 2 cm, which could be treated with definitive primary bone grafting, it is important to consider that the management of soft tissues is often the determining factor for treatment success. In the patients included in this review, a concomitant infection would have likely led to unsatisfactory outcomes if direct bone grafting had been chosen rather than deploying the Masquelet induced membrane technique.

Another interesting finding is the confirmation that the size of the bone defect does not significantly correlate with the time required for its healing, which averages around 7 months. This value is extremely competitive when compared with other therapeutic strategies. Distraction osteogenesis requires about 10 months for defects smaller than 5 cm and about 20 months for defects between 5 and 10 cm [31]. Although new studies aim to reduce the time associated with this procedure [32], the difference between the two techniques remains significant in terms of both duration and required patient compliance [33]. Another alternative is vascularized fibula grafting [34], which could reduce the bone union time by 1.5–2 months. However, this technique has several limitations: the need for a much longer surgical procedure and the requirement for microsurgical expertise [35]. Additionally, the reoperation rate exceeds 30%, with a non-union incidence of 17% [36].

It is not surprising that infection was the most frequently described complication in this study (6%). As Masquelet emphasized [37], this underscores the importance of radical debridement during the first phase of the procedure. Infection eradication primarily depends on surgical debridement rather than on the antibiotic added to the spacer or on the increased local vascularization induced by the membrane [38].

The causes underlying the failure of bone consolidation observed in 6 patients (4%) are unclear. Possible contributing factors include an ongoing infectious process, non-viable soft tissues, insufficient local mechanical stability, and inadequate technique in filling the bone defect [39]. In these cases, a multidisciplinary approach is essential. Collaboration between an infectious disease specialist, plastic surgeon, vascular surgeon, and internist optimizes patient conditions, creating a sterile, vascularized, and stable environment that, with the intervention of an experienced orthopedic surgeon, can promote bone union.

Research on the induced membrane technique is still in its early stages, and many questions remain unanswered. Future studies, ideally prospective and multicentric, are needed to fully understand the biology of the induced membrane and identify aspects worth modifying to achieve better outcomes and fewer complications in both the lower and upper limbs. In the immediate term, standardizing the definition of bone union would reduce bias in published data and, consequently, in their analysis.

This study presents several limitations. The first concerns the low quality and limited level of evidence of the included studies, with sample sizes not exceeding 10 units in more than 50% of cases and an average MINORS score of 9. A second limitation is the heterogeneity of the patients, ranging from adults with open fractures to pediatric patients with oncological conditions. A third limitation is the variability of the data provided by the authors and the lack of a clear and consistent definition of bone union. Finally, an additional limitation is the lack of data concerning soft-tissue compromise, which is one of the key determining factors in bone healing.

## Conclusions

In conclusion, the induced membrane technique represents a viable and effective option for managing segmental bone defects in the upper limb. Its ability to handle large defects and its relatively straightforward application make it an attractive alternative to more complex methods. However, paying attention to potential complications and continued research into optimizing surgical practices are crucial for maximizing the technique’s benefits and improving patient outcomes.

## Data Availability

Not applicable.
